# Feasibility of a custom-tailored, evidence-based, theory-informed, intervention to prevent burnout and reduce stress for healthcare professionals: protocol for a single-arm trial

**DOI:** 10.1186/s40814-024-01553-w

**Published:** 2024-11-07

**Authors:** Marleen Schröter, Julia Berschick, Anna K. Koch, Julia K. Schiele, Martin Bogdanski, Melanie Steinmetz, Wiebke Stritter, Christian S. Kessler, Georg Seifert

**Affiliations:** 1 Department of Prevention, Integrative Medicine and Health Promotion, Charité–Universitätsmedizin Berlin, Corporate Member of Freie Universität Berlin, Humboldt-Universität Zu Berlin, and Berlin Institute of Health, Berlin, Germany; 2 Institute of Social Medicine, Epidemiology and Health Economics, Charité–Universitätsmedizin Berlin, Corporate Member of Freie Universität Berlin, Humboldt-Universität Zu Berlin, and Berlin Institute of Health, Berlin, Germany; 3 Department of Internal Medicine and Nature-Based Therapies, Immanuel Hospital Berlin, 14109 Berlin, Germany

**Keywords:** Burnout, Prevention, Healthcare professionals, Study protocol, Feasibility, Mind–body medicine

## Abstract

**Background:**

Healthcare professionals face high levels of occupational stress, time pressure, workload, and poor organizational support. This makes them particularly vulnerable to burnout. The COVID-19 pandemic has further exacerbated this situation. This single-arm, multicenter, mixed-methods feasibility study pilots the *LAGOM* program: A tailored, evidence-based intervention to prevent burnout and reduce stress among healthcare professionals.

**Methods:**

Participants will include healthcare professionals (*N* = 30) working at Charité–Universitätsmedizin Berlin and Immanuel Hospital, Berlin. *LAGOM* focuses on support for individual behavior change and personal resources and also addresses the organizational level. The intervention´s feasibility will be evaluated through a non-randomized feasibility trial with a mixed methods process evaluation. The exploratory primary study aims are to assess the acceptability and feasibility of the (1) evaluation procedures and of the (2) intervention content and structure using study records, standardized questionnaires, protocol checklists, and diaries. Exploratory effectiveness analysis will take place as well. Further, semi-structured interviews (*n* = 3 to 6) and electrophysiological measurements (*n* = 20) will be conducted.

**Discussion:**

Custom-tailored, well-implemented multi-level interventions are needed to prevent burnout and reduce stress among healthcare professionals. Long-term strategies are warranted to sustainably implement effective programs. This feasibility study helps to refine trial procedures and content of the *LAGOM* program for a randomized controlled trial to evaluate the intervention’s effectiveness.

**Trial registration:**

German Clinical Trials Register: DRKS00032014, registered 17^th^ October 2023.

**Supplementary Information:**

The online version contains supplementary material available at 10.1186/s40814-024-01553-w.

## Background

Burnout in healthcare professionals is a global problem with both negative health consequences for the individual and negative effects on patient safety, patient care, professionalism, workplace injuries, and absenteeism [1, 2]. The COVID-19 pandemic has exacerbated the situation [3, 4]. Effective interventions to prevent burnout in healthcare professionals are more urgent than ever. Due to the unique and dynamic work environment where healthcare professionals face high levels of occupational stress, time pressure, workload, and, more often than not, poor organizational support [5], the implementation of effective interventions in this context is particularly challenging.

Burnout is a multidimensional construct, often defined by the symptom triad of (1) emotional exhaustion, (2) depersonalization, and (3) reduced personal accomplishment [6]. As individual factors, as well as factors of the working context, play a role in the development of burnout, interventions to address burnout should incorporate both aspects [6]. Both individually oriented and structural strategies have the potential to lead to a clinically significant reduction in burnout among physicians and nurses [7, 8], at best combining both approaches to be as successful as possible [9]. Yet most interventions focus on either one or the other: Either person-directed courses offering a combination of mindfulness, self-care, yoga, massage, meditation, or stress management skills or organization-directed interventions like workload or schedule rotations [8]. Very few interventions combine both approaches [10, 11]. Another significant pitfall of ineffective interventions is the lack of explicitly tailoring the interventions to the specific needs of healthcare professionals and their organizations [12]. This can lead to unintended side effects or low participation, subsequently resulting in no improvements in health outcomes [12]. In addition, assessments of intervention adherence are scarce [12]. This limits the ability to assess whether the intervention was not properly implemented or was simply ineffective [7, 12, 13].

This paper describes the protocol for the *LAGOM* feasibility study. *LAGOM* is a Swedish word describing the “golden ratio”, if something is just right, not too much and not too little, the ideal equilibrium. It is also an acronym for “LAngfristig Gesundheitsbezogene Organisationskonzepte mit Mind–Body Medizin” (Longterm Approach and Guidelines for Occupational Mental health with Mind–Body Medicine). It is a custom-tailored, evidence-based, theory-informed intervention to prevent burnout and reduce stress for healthcare professionals. *LAGOM's* long-term goal is to create a sustainable, health-promoting, and meaningful work environment in hospitals so that work is enjoyable, employees experience job satisfaction, and remain healthy in the long term. The intervention was developed in close collaboration with healthcare professionals throughout the process following the Intervention Mapping Approach (IMA) [14]. *LAGOM* focuses on both individual and structural prevention, precisely addressing the known weaknesses of existing burnout prevention interventions for healthcare professionals [7, 13]. Individualized prevention contains elements of mind–body medicine (MBM), a health practice that combines mental focus, breathing exercises, and body movements to calm down body and mind and promote health and well-being [15]. It encompasses a wide variety of techniques such as meditation, yoga, or guided imagery, and research has proven that there are beneficial effects on multiple physical and mental health conditions related to stress [16, 17]. Due to the complex processes and costs associated with implementing and evaluating such sophisticated behavior change interventions for health professionals, piloting the intervention prior to a confirmatory randomized controlled trial (RCT) is essential.

This feasibility study has two main objectives:

1. Assess the feasibility and acceptability of trial and evaluation procedures (e.g. recruitment rates).
2. Assess the feasibility and acceptability of the *LAGOM* content and structure (e.g. satisfaction with the *LAGOM program*).

## Methods

### Study setting and design

**Fig. 1:**
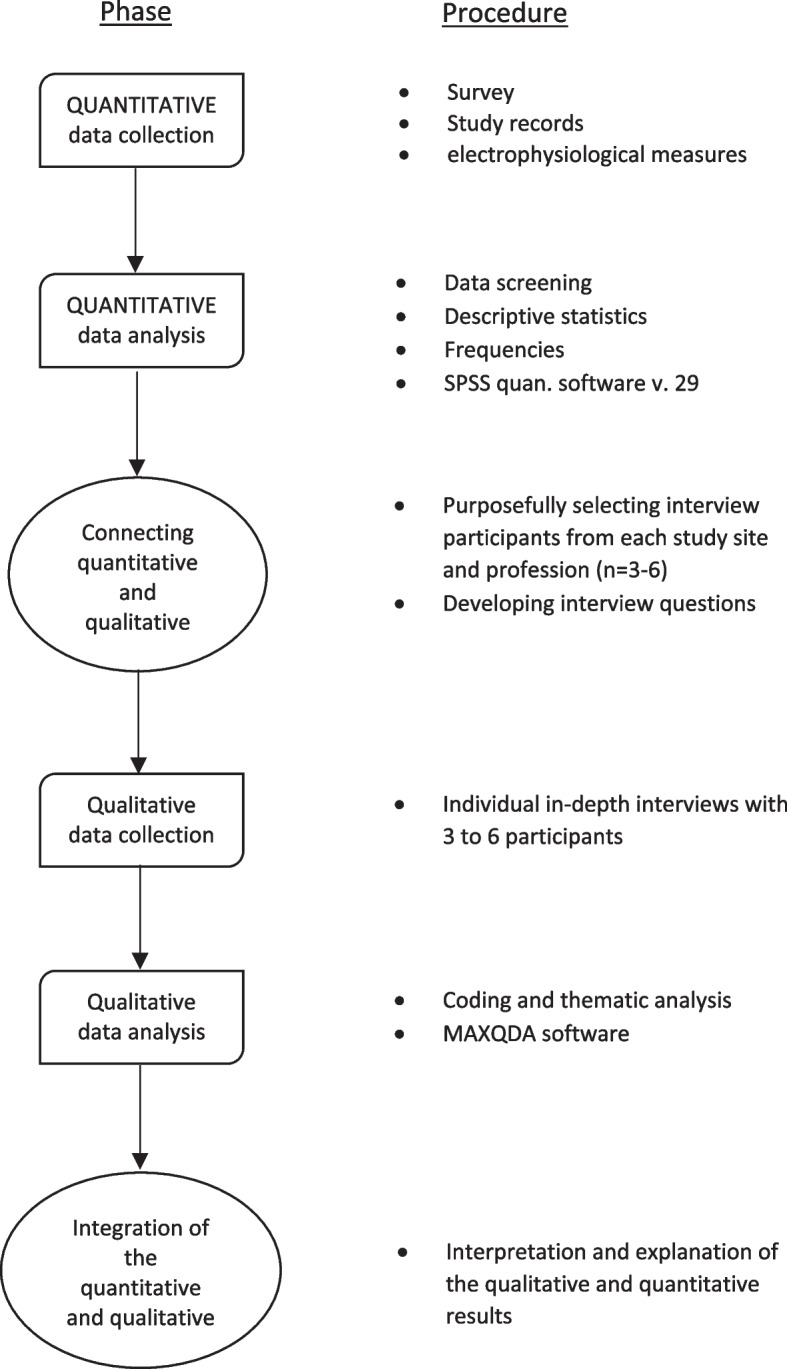
Mixed methods sequential explanatory design flowchart, adapted from Ivankowa, Creswell & Stick, 2006, p. 16 [23]

The single-arm, multi-center, mixed-methods feasibility trial will be conducted and reported according to the SPIRIT guideline [18], supplemented by the CONSORT extension to pilot and feasibility trials [19], the Mixed-Methods Article Reporting Standards [20] and the IMA [14]. It received favorable ethical approval from the Ethics Committee of the Charité–Universitätsmedizin Berlin on 14th July, 2023 (EA1/157/23) and has been registered in the German Clinical Trials Register prior to conducting the study (DRKS00032014). The research will adhere to standards of good clinical practice and the declaration of Helsinki. Study sites are the Charité–Universitätsmedizin, Berlin and the Immanuel Hospital, Wannsee, Germany. A mixed methods sequential explanatory design will be used which consists of two phases, a quantitative phase, followed by a qualitative phase [21] as depicted in Fig. 1. First, quantitative data is collected and analyzed. In the second step, qualitative data is collected and analyzed to follow up on the experiences of participants and elaborate on the quantitative results. The qualitative phase builds upon the quantitative phase and the two phases are connected at the intermediate stage of the study as well as at the interpretation stage. This mixed-methods approach enables the quantitative data and analysis to provide a general understanding of the feasibility of the intervention. Subsequently, the qualitative data and analysis will follow up on the experiences of participants with the intervention by exploring participants´ views in more depth and help refine and explain quantitative data [22].

### Eligibility criteria

Healthcare professionals working at Charité–Universitätsmedizin Berlin or Immanuel Hospital, Wannsee, Germany will be invited to participate. Table 1 outlines the inclusion and exclusion criteria.

**Table 1:** Eligibility criteria

Inclusion criteria
• Working Healthcare professionals, actively practicing medicine or nursing at Charité–Universitätsmedizin Berlin or Immanuel Hospital, Wannsee, Germany • 18 years or older • Completed written informed consent • Proficient in the German language
Exclusion criteria
• Clinically diagnosis of burnout syndrome according to ICD-11 (QD85 “Burnout”) • Pregnancy • Solely administrative position

### Recruitment procedure

Participants for the *LAGOM* program will be recruited by the research team via the hospitals´ intranet, notices, and informational events, as well as by word of mouth at Charité–Universitätsmedizin Berlin and Immanuel Hospital. The management supports the participation of their employees by scheduling time off for course hours. Eligible participants will be informed about the study process and will receive written study information to take home and read at their convenience. A few days later, they will be contacted via telephone by a member of the study team and asked if they would like to participate. During telephone contact, participants will be given the opportunity to ask questions to clarify any uncertainties. Interested participants will then provide written informed consent and complete the baseline questionnaire to be enrolled in the study. Reasons for not granting consent or completing the baseline survey will be recorded. All participants will be explicitly informed that they can withdraw from the study at any time without giving reasons and that this will not have any negative consequences for their jobs.

### Intervention

#### Intervention development

The intervention development followed the IMA by Eldredge et al. (2006) [14]. In addition to the project team, which consists of physicians, psychologists, nutritionists, sports scientists, and physical medicine specialists, various advisory boards were involved in intervention development. A group of stakeholders, consisting of physicians and nurses, the target group of the intervention, also formed part of the expert advisory boards. This stakeholder expert advisory board gave continuous feedback during the intervention development phase and this assessment informed and tailored the program specifically to ensure that the needs of the target group will be addressed. The *LAGOM* program and the IMA steps will be described in detail in a subsequent publication. In the following, the content and structure of the *LAGOM* program are briefly outlined.

### Individual LAGOM program aspects

The *LAGOM* program will take place over a period of 9 weeks with one session per week. The first and last sessions will last two hours to provide enough time for introduction, group familiarization, and time to review the learned concepts, give a future perspective, and course conclusion. The sessions will last 90 min to enable facilitation during working hours. Meetings will alternate between in-person and online sessions to provide participants more flexibility to take part in the course. The sessions follow the same structure: (1) Introduction with activating movement exercise, observation of a minute of silence for centering after arrival, and reflection (2) psycho-educational part on different topics (see Table 2) with practical exercises and group exchange, (3) a relaxation exercise, (4) session conclusion. Topics have been derived from the needs assessment, complemented with elements from mind–body medicine, such as breathing exercises, various meditation practices, yoga exercises, acupressure, mindful walking, progressive muscle relaxation, imagination exercise, self-reflective exercises, and more. In addition, in-depth material such as self-reflection exercises, guided meditations, or literature resources will be provided and participants will be encouraged to practice 5–15 min daily at home or work. The weekly *LAGOM* sessions will be conducted by mind–body-educated and experienced trainers. A short description of the topics and content that is covered is provided in Table 2.

**Table 2:** The LAGOM program: an overview of the session topics, components, and organizational aspects

Session	Topic	Components (examples)	Organizational impulses
1	Introduction to the LAGOM program	• Getting to know each other • Introduction to the “Temple of health” concept • Phases of Behavior change • Goal setting	• Information on already existing occupational health services • Posters and calendars on health topics as visual reminders for hospital wards
2	Stress and stress patterns	• identifying stress patterns • Burnout definition and prevention • Resilience measures/activities/resources	• “Open Ear Policy”: appointment with clinic management to raise concerns and needs
3	Healthy routines in shift work	• Identifying break habits • Learning self-care strategies	• Tips and support for healthy breaks and break room makeovers • Tips to establish daily healthy snacks for wards
4	Cognitive regulation and power of thoughts	• Introduction of the ABC-D concept	• Guidelines for a mental health check-up as part of employee appraisals
5	Managing and developing the “inner team”	• Introduction of the “inner team” concept • identifying needs	• Short massage opportunity at the workplace
6	(Future-)Values in work culture	• Ecosystem clinic, inspired by theory U • Practicing social body scan	• Team coaching/supervision within ward team
7	Communication with others	• Development of empathic, active listening • Introduction of the “compass of needs” idea • Introduction of the stressor identification of 4-ear-model	• Conflict resolution within ward team with external mediation
8	Balance between self-care and care for others	• Protection and mindful use of own resources • Identification of Self-care and self-compassion resources	• Introduction of periodical interdisciplinary “happy hour” lunch breaks
9	“The end of the course is the beginning of…?”—Outlook	• Concept review • Celebrating successes • Conclusion	• Forest therapy sessions as team building for wards of study participants

#### Structural and organizational LAGOM program aspects

In addition, in dialog with the individual hospital wards, structural and organizational aspects will be addressed weekly as well (Table 2). Participants will be able to choose suitable activities according to the different needs and prerequisites of their wards and teams.

### Outcome measures

Data will be collected before (week 0), during (week 1–9) and after the trial (week 10) to assess the acceptability and feasibility of the trial procedures and intervention content. Data will be assessed via SoSci Survey, a web application for online surveys, as well as semi-structured interviews. Data on SoSci is collected and pseudonymized so that an assignment of baseline questionnaires to post-questionnaires but no identification of individuals is possible. Also, the electrophysiological measure analyses planned for the RCT following the feasibility study will be piloted within this study. Table 3 outlines the schedule of enrollment, data collection, and outcome measures.

**Table 3:** Feasibility evaluation plan for the *LAGOM* Program

			Study period			
	Measures	Sources	Pre-Intervention	Baseline	*LAGOM*- program	Post-Intervention
				Week 0	Week 1–9	Week 10
Enrollment						
Eligibility screen	Eligibility checklist	Participants	X			
Informed consent and assent		Participants	X			
Data collection						
Sociodemographics	Survey	Participants		X		
Acceptability and feasibility evaluation (*N* = 30)						
Trial procedures						
Recruitment	Study records	Research staff	X			
Attrition	Study records	Research staff			X	X
Completeness of data collection	Study records	Research staff		X		X
Assessment process	Survey	Participants				X
Protocol adherence	Protocol checklist	Trainers, Research staff			X	
Intervention adherence	Study records	Trainers			X	
Intervention content and structure						
Satisfaction	Q4TE	Participants				X
Utility	Q4TE	Participants				X
Knowledge	Q4TE	Participants				X
Application to practice	Q4TE	Participants				X
Organizational results: individual	Q4TE	Participants				X
Organizational results: global	Q4TE	Participants				X
Safety	Study records	Trainers Participants			X	X
Perceived fit and recommendations	Survey	Participants				X
Effectiveness evaluation (exploratory)^a^ (*N* = 30)						
Quality of life						
Burnout symptoms	Questionnaire (MBI)	Participants		X		X
Behavior						
Break habits	Diaries	Participants			X	
Environmental conditions						
Open ear appointment	Email/Phone Call	Participants			X	
Determinants						
Self-efficacy	Questionnaire (BSW-5-REV)	Participants		X		X
Semi-structured interviews (*n* = 3 to 6)						
Perceived barriers	Interviews	Participants				X
Perceived support by supervisor(s)	Interviews	Participants				X
Perceived support from colleagues	Interviews	Participants				X
Perceived benefit on mental health	Interviews	Participants				X
Consolidation	Interviews	Participants				X
Perceived effect on the work environment	Interviews	Participants				X
Recommendations	Interviews	Participants				X
Electrophysiological measures (*n* ≥ 20 with ≥ 10 at each site)						
Accessibility and eligibility of the locality/lab	Survey	Participants		X		
Schedule management	Survey	Participants		X		
Measurement procedure and duration	Survey	Participants		X		
Example HRV estimation based on SDNN and RMSSD	Electrophysiological measures	Participants		X		X

*Q4TE* Questionnaire for Professional Training Evaluation, *MBI* Maslach Burnout Inventory, *BSW-5-Rev* Scale for measuring occupational self-efficacy expectation [Skala zur Messung der beruflichen Selbstwirksamkeitserwartung], *HRV* Heart rate variability, *SDNN* Standard deviation of all NN (heartbeat) intervals, *RMSSD* Square root of the mean squared differences of successive NN intervals

^a^Like the development of the *LAGOM* program, the effect evaluation will be based on Eldredge’s IMA; accordingly, a consideration of the four main components in IMA (quality of life, behavior, environmental conditions, and determinants) is planned

### Demographic data

Sociodemographic variables include age, gender, height, weight, occupation, full-time yes/no, cultural background (optional), and shift work yes/no.

### Acceptability and feasibility evaluation

#### Trial procedures

*Recruitment* is defined as the number (*n*) of participants who were sent a participant information sheet, number who agreed to participate, and number recruited per week. It will be assessed by the research team via study records. *Attrition* is defined as participant dropout over time, recorded by the study team in the study records. Reasons for dropout will be documented for participants who consent to provide a reason but clearly stipulate that giving a reason for study withdrawing is not mandated. *Completeness of data collection* is defined as the recorded number of completed surveys returned to the trial team at each measurement point. It will be assessed by the research team via study records. The *assessment process* will be evaluated via quantitative survey data with regard to comprehensibility, accessibility, and time management. The following statements can be rated on a five-point response scale ranging from 1 = *totally disagree* to 5 = *totally agree*: (1) the survey questions were comprehensible; (2) access to the questionnaires was easy; (3) the time required for completion of the surveys was compatible with my daily work routine; (4) the documentation effort (break behavior, adverse events) was compatible with my daily work routine. *Protocol adherence* is defined as the degree to which the intervention was implemented as prescribed in the protocol, measured through protocol checklists completed by trainers and research staff. *Intervention adherence* is defined as the number of sessions attended, and documented by the trainers.

#### Intervention content and structure

In order to evaluate intervention acceptability, usefulness, participants´ learning, implementation, and transfer, the Questionnaire for Professional Training Evaluation (Q4TE), a validated training evaluation questionnaire [24] will be used. The questionnaire consists of six subscales (*Satisfaction, Utility, Knowledge, Application to practice, Individual organizational results, and Global organizational results*) with 12 items that can be rated on an 11-point response scale ranging from 0 percent = *completely disagree* (coded as 0) to 100% = *completely agree* (coded as 10). For item 11 (“Overall, it seems to me that the application of the training contents has facilitated the workflow in my company.”) the expression “my company” was changed to “my hospital unit” to more accurately represent the study setting. Previous research has shown that the Q4TE has good discriminant validity and internal consistency (Cronbach’s α = 0.79 to 0.96) [24]. *Safety* is defined as intervention-related adverse events. Safety will be assessed through study records by trainers and participants. *Perceived fit and recommendations* will be further assessed by quantitative survey data and open questions to allow more in-depth information on participants´ experiences with the *LAGOM* program. This will include the following items:

1. The time required to participate in the *LAGOM* program was compatible with my daily work routine (five-point response scale, 1 = *totally disagree* to 5 = *totally agree*).
2. The following course time was most compatible with my daily work routine: (drop-down menu of answers)
3. The group size was appropriate (five-point response scale, 1 = *totally disagree* to 5 = *totally agree*).
4. Would you recommend the *LAGOM* program to other employees? (yes/no and open field for reason)
5. Would you recommend the trainer to other employees? (yes/no and open field for reason)
6. What was particularly helpful about the *LAGOM* program? (open question)
7. What would you recommend changing about the *LAGOM* program? (open question)

### Exploratory effectiveness evaluation

Since this is a feasibility study without sample size calculation, effectiveness measurements are made purely exploratory to evaluate the feasibility of questionnaires and assessment approaches. Like the development of the *LAGOM* program, the effect evaluation will be based on the IMA, which is why a consideration of the four main components in IMA (quality of life, behavior, environmental conditions, and determinants) is planned [14]. *Quality of Life*: Burnout symptoms will be assessed by the Maslach Burnout Inventory, the German version. The MBI addresses three subscales (Emotional Exhaustion [EE], Depersonalization [DP], and Personal Accomplishment [PA]) with 22 items that can be rated on a scale ranging from 0 = never to 6 = every day with higher scores indicating a higher level of burnout for EE and DP and lower scores indicating a higher level of burnout for PA. Validity and reliability of the MBI have been demonstrated to be good or acceptable (Cronbach´s α > 0.7 for all subscales) [25–27]. *Behavior*: changes in frequency and duration of taking breaks will be assessed daily through a diary, documented by participants throughout the program period. *Environmental conditions*: implementation of open ear appointments (whether they have taken place or not) will be assessed as an indicator of the environmental determinant. Information will be gathered by the research staff from participants via email or phone call. *Determinants*: occupational self-efficacy will be assessed by the BSW-5-Rev (German: Skala zur Messung der beruflichen Selbstwirksamkeitserwartung; scale for measuring work-related self-efficacy) [28]. It consists of 5 items that can be rated on a scale ranging from 1 = *completely disagree* to 4 = *completely agree,* indicating how far the statement applies to oneself or not. Research has shown good construct and criterion validity and acceptable internal consistency (Cronbach’s α = 0.73 for the employee version) [28].

### Semi-structured interviews

Semi-structured interviews will be conducted with three to six intervention participants to further explore their experience with the intervention. They will be selected strategically to represent a wide range of experience from the two disciplines, hospital units, and study sites. Questions selected for the interview will be based on understanding the complexity of experience, and topics that may have sensitive content or that arise from the questionnaires or course content. The interviews will be based on the following questions:

- What difficulties or obstacles did you experience in participating in the training?
- How did you perceive the support from your supervisor(s)? What did you feel supported by? What did you not feel supported by?
- How did participation in the training impact you personally?
- How was your participation perceived by your colleagues?
- Is there something that you would find helpful now that you have completed the training?
- What other ideas for the program do you have to promote a healthy working atmosphere?
- Finally, would you like to give us some advice for the finalization of the program?

Some results of the survey data may require an in-depth follow-up making additional interview questions necessary that cannot be stated a priori but are rather generated by survey data.

### Electrophysiological measures

The investigation of electrophysiological measures from which the autonomic regulation and the sympathovagal balance can be derived is a suitable method to evaluate the progress of the individual physical (stress-) state or the effect of a balancing and resilience-strengthening intervention [17, 29–31]. The electrophysiological recordings will be conducted at baseline and after the end of the intervention using a Somnomedics SOMNO HD electrophysiological measure recording system.

Electrophysiological measures will be derived from at least ten intervention participants at each study site. The main focus is to test whether the procedures of conducting electrophysiological recordings are feasible within a future trial rather than collecting complete data. Participants can sign up independently in a booking list with predefined appointments. The measurements are carried out by trained specialist staff in a quiet room on the clinic campus. A maximum of up to 30 min is planned for one measurement appointment. After a welcome and brief explanation of the procedure, the non-invasive measurement equipment is applied to the study participant, who is asked to sit quietly and move as little as possible during the recording. Movements and talking would otherwise impair the quality of the recorded signals and make post-processing even more necessary or completely unusable. The recording should provide at least 15 min of high-quality signals in a continuous session.

The feasibility of the electrophysiological measure analysis will be assessed at baseline by the following questions that can be rated on a five-point response scale ranging from 1 = *totally disagree* to 5 = *totally agree*:

1. The time required for the electrophysiological measures was compatible with my daily work routine.
2. The arrangement of the appointment for the electrophysiological measures was easy to make.
3. The location for the electrophysiological measures was easy to find.

The following electrophysiological measures will be derived both at baseline and post-intervention in a resting seated position:

1. Common electrophysiological measures monitoring device: electrocardiography (3-channel ECG), respiration activity (respiration-belt), pulse wave (finger clip PPG/photoplethysmography), and electrodermal activity (EDA, finger clips).
2. Wearable (bracelet): pulse (heart rate), pulse wave (PPG), derivative respiration activity, and EDA.

The ECG records the heart’s activity and enables the time of each heartbeat to be registered. In order to analyze HRV, the times between the registered heartbeats are plotted in a tachogram (time series of all RR / beat-to-beat intervals, usually expressed in milliseconds). This RR series is then corrected for technical or physiological disturbances using different signal processing methods (such as filtering and artifact correction), resulting in an NN series (normal-to-normal time series). On the basis of this NN series, numerous HRV parameters can then be determined. The blood volume pulse derived from a photoplethysmography fingerclip (also used for pulse oximetry) allows for the registration of blood volume changes in the small vessels of the fingertip. This method also offers the possibility of registering heartbeats and calculating their beat-to-beat time intervals. HRV analyses are therefore also possible in this way in order to obtain cardiac information on the condition of the autonomic nervous system (ANS). However, vascular information can also be derived from the amplitude changes of this pulse wave signal, which provides additional information on the ANS status. The respiration belt adds another dimension to the ANS functioning. By stretching and compressing the belt during inhalation and exhalation, the breathing activity can be determined. This provides information about the duration and depth of the inspiration and expiration as well as the breathing rate. Finally, the measurement of the EDA yields information about the skin conductance (on the hands), which is sensitive to sweat. This gives a further dimension for estimating the ANS and can also serve as a stress marker. The measurements are also accompanied by simplified derivations using wearables. This is primarily intended to test the handling of these devices. In addition, it should then be possible to check internally whether these mobile devices alone would be qualitatively sufficient for evaluations in the future. These results may not be reported.

The selected electrophysiological measures are representatives of the state of autonomic regulation. In the feasibility study, the focus will be on the implementation and process for data collection of the electrophysiological measures. Since this is the main concern of this feasibility study, only two example HRV indices measures will be calculated from the recorded data. Based on these, the general calculation procedure of all future variability measures will be verified. The two standard HRV measures involved here are SDNN and RMSSD. SDNN is defined as the standard deviation of all NN (normal-to-normal heartbeat) intervals as a measure for total HRV while RMSSD as the square root of the mean squared differences of successive NN intervals reflects the short-term HRV. These are exemplary of a comprehensive assessment of the intervention effect on autonomic regulation in the following RCT. This future investigation will include numerous indices from HRV (cardiac activity function from ECG or PPG heartbeats), pulse wave variability (PWV, vascular variability function from PPG), EDA, and respiratory activity using different methods of electrophysiological measure analysis in time and frequency domains as well as non-linear dynamics.

### Data analysis

#### Quantitative

**Fig. 2:**
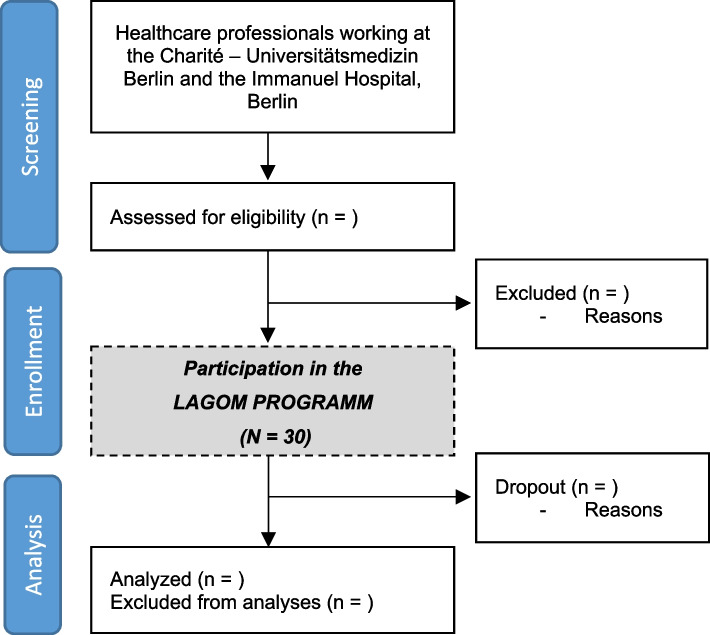
Feasibility trial flow diagram

A CONSORT flowchart (Fig. 2) will provide information on the number of participants screened, enrolled, and analyzed. Reasons for dropout during the intervention will be recorded and reported. A table showing baseline demographic and clinical characteristics will be presented. Adverse events will be reported. Since no confirmatory hypotheses are to be tested within the present pilot study, all exploratory effectiveness outcomes are evaluated and presented purely descriptively using means, medians, standard deviations, minimum, maximum, and percentages as appropriate. The electrophysiological measure analysis will include calculations of two standard HRV measures (SDNN and RMSSD) from at least 20 participants. All analyses will be performed using the Statistical Package for Social Sciences software (IBM SPSS Statistics for Windows, release 29.0; IBM Corporation, Armonk, NY) and MathWorks MATLAB (MATLAB version: 9.13.0 (R2022b), Natick, MA: The MathWorks Inc.; 2022).

#### Qualitative

Data will be analyzed according to the qualitative content analysis by Kuckartz (2018) [32] assisted by the qualitative and mixed methods research software MAXQDA 2022.

#### Integration of quantitative and qualitative results

Quantitative and Qualitative results will be integrated at the interpretation stage as well [22], by triangulating and qualitatively exploring contradictions, explanations, or experiences in-depth that arise from the quantitative data.

#### Transition to a future pragmatic trial

Based on the results of this pilot study, the project team, together with the other stakeholders involved in the project, will critically discuss potentially necessary program modifications in the run-up to the pragmatic trial. The criteria defined in Additional file 1: Table S1 in the additional file serve as the basis for this discussion.

#### Sample size

Since this is a feasibility study for which it is not necessary to adequately power for statistical null hypothesis testing, no formal sample size calculation was made a priori [33]. A fixed number of *N* = 30 was chosen, based on practical considerations and recommendations for good practice in pilot studies [34]. For the second qualitative phase, a strategic sub-set of three to six participants will be chosen to explore a wide range of experiences from the two disciplines, hospital units, and study sites. A minimum number of *n* = 20 with at least 10 participants from each site was selected as appropriate for evaluation of the electrophysiological measure procedures. Depending on feedback and schedule management, attempts will be made to include more participants.

## Discussion

This paper describes the protocol for the *LAGOM* feasibility study. *LAGOM* is a tailored, evidence-based, theory-driven burnout prevention and stress reduction intervention for healthcare professionals developed according to the IMA [14]. Burnout among healthcare professionals is a global problem with negative consequences for the individual but also for the professional environment [1, 2]. The COVID-19 pandemic has aggravated the situation [3, 4]. Because of the particularly stressful and dynamic environment in healthcare, developing and implementing effective burnout prevention interventions in this context is particularly challenging. Hospitals in Germany are compelled by law to offer effective preventive measures in this sector. These must not be based exclusively on individual prevention, because burnout is a multidimensional construct that is mostly caused by poor working conditions. Structural prevention should always be part of the prevention offers with the aim of a sustainable structural improvement of working conditions for healthcare professionals. The *LAGOM* intervention development was done in close collaboration with healthcare professionals throughout the process, targets both individual and structural prevention, and thus addresses precisely the known weaknesses of existing interventions for burnout prevention for healthcare professionals. The long-term aim is to create a sustainable working environment at hospitals that is conducive to health and meaning, so that the job is enjoyable, and that employees experience job satisfaction and stay healthy on a long-term basis. Due to the complex processes and costs associated with implementing and evaluating such sophisticated behavior change interventions for healthcare professionals, piloting the intervention and the evaluation plan prior to an efficacy study in a pragmatic RCT is essential. The goal of the study is to assess the feasibility and acceptability of the intervention from the perspective of participants, trainers, supervisors, and research staff. This provides an opportunity to reveal problems in practical implementation and to fine-tune the program.

## Supplementary Information

Additional file 1: Table S1. Trial outcomes and modification criteria-basis for the discussion regarding potential modifications for the future pragmatic trial. The table defines success criteria for intervention outcomes which will serve as a basis for the discussion.

## Data Availability

Not applicable.

## Abbreviations

BSW-5-Rev: Scale for measuring occupational self-efficacy expectation [Skala zur Messung der beruflichen Selbstwirksamkeitserwartung]
DP: Depersonalization
EE: Emotional exhaustion
HRV: Heart rate variability
IMA: Intervention mapping approach
MBI: Maslach Burnout Inventory
NN: Normal-to-normal heartbeat
PA: Personal accomplishment
PWV: Pulse wave variability
Q4TE: Questionnaire for Professional Training Evaluation
RCT: Randomized controlled trial
RMSSD: Root mean square of successive differences (precisely the square root of the mean squared differences of successive NN intervals)
SDNN: Standard deviation of all NN intervals

## References

[1] Wright T, Mughal F, Babatunde OO, Dikomitis L, Mallen CD, Helliwell T. Burnout among primary health-care professionals in low-and middle-income countries: systematic review and meta-analysis. Bull World Health Organ. 2022;100(6):385. 35694622 10.2471/BLT.22.288300 PMC9178426

[2] Hodkinson A, Zhou A, Johnson J, Geraghty K, Riley R, Zhou A, et al. Associations of physician burnout with career engagement and quality of patient care: systematic review and meta-analysis. BMJ. 2022;378:e070442. 36104064 10.1136/bmj-2022-070442 PMC9472104

[3] Azoulay E, De Waele J, Ferrer R, Staudinger T, Borkowska M, Povoa P, et al. Symptoms of burnout in intensive care unit specialists facing the COVID-19 outbreak. Ann Intensive Care. 2020;10(1):1–8. 32770449 10.1186/s13613-020-00722-3 PMC7414284

[4] Morgantini LA, Naha U, Wang H, Francavilla S, Acar Ö, Flores JM, et al. Factors contributing to healthcare professional burnout during the COVID-19 pandemic: a rapid turnaround global survey. PLoS One. 2020;15(9):e0238217. 32881887 10.1371/journal.pone.0238217 PMC7470306

[5] Dugani S, Afari H, Hirschhorn LR, Ratcliffe H, Veillard J, Martin G, et al. Prevalence and factors associated with burnout among frontline primary health care providers in low-and middle-income countries: a systematic review. Gates Open Res. 2018;2:4. 29984356 10.12688/gatesopenres.12779.3 PMC6030396

[6] Berger M, Linden M, Schramm E, Hillert A, Voderholzer U, Maier W. Positionspapier der Deutschen Gesellschaft für Psychiatrie, Psychotherapie und Nervenheilkunde (DGPPN) zum Thema Burnout. Nervenarzt. 2012;4:537–43.

[7] West CP, Dyrbye LN, Erwin PJ, Shanafelt TD. Interventions to prevent and reduce physician burnout: a systematic review and meta-analysis. Lancet. 2016;388(10057):2272–81. 27692469 10.1016/S0140-6736(16)31279-X

[8] Zhang XJ, Song Y, Jiang T, Ding N, Shi TY. Interventions to reduce burnout of physicians and nurses: an overview of systematic reviews and meta-analyses. Medicine (Baltimore). 2020;99(26):e20992. 32590814 10.1097/MD.0000000000020992 PMC7328917

[9] Maslach C, Leiter MP. Understanding the burnout experience: recent research and its implications for psychiatry. World Psychiatry. 2016;15(2):103–11. 27265691 10.1002/wps.20311 PMC4911781

[10] Schiele JK, Koch AK, Adam D, Berschick J, Schröter M, Reschke S, Stritter W, Blakeslee S, Sehouli J, Seifert G, Kessler CS. Vom Burnout zur Balance. Programme in deutschen Krankenhäusern: Graue Literaturübersicht mit semi-strukturierten Interviews (from burnout to balance. Programs in German hospitals: grey literature review with semi-structured interviews). Zeitschrift Arbeitsmedizin Sozialmedizin Umweltmedizin. 2023;59:38–45.

[11] Adam D, Berschick J, Schiele JK, Bogdanski M, Schröter M, Steinmetz M, et al. Interventions to reduce stress and prevent burnout in healthcare professionals supported by digital applications: a scoping review. Front Public Health. 2023;11:1–10. 10.3389/fpubh.2023.1231266 PMC10630920 38026413

[12] Dreison KC, Luther L, Bonfils KA, Sliter MT, McGrew JH, Salyers MP. Job burnout in mental health providers: a meta-analysis of 35 years of intervention research. J Occup Health Psychol. 2018;23(1):18. 27643608 10.1037/ocp0000047

[13] Swensen S, Kabcenell A, Shanafelt T. Physician-organization collaboration reduces physician burnout and promotes engagement: the Mayo Clinic Experience. J Healthc Manag. 2016;61(2):105–27. 27111930

[14] Eldredge BLK, Markham CM, Ruiter RAC, Fernández ME, Kok G, Parcel GS. Planning health promotion programs: an Intervention Mapping approach. 4th ed. San Francisco: Jossey-Bass Inc.; 2016.

[15] Astin JA, Shapiro SL, Eisenberg DM, Forys KL. Mind-body medicine: state of the science, implications for practice. J Am Board Fam Pract. 2003;16(2):131–47. 12665179 10.3122/jabfm.16.2.131

[16] Weinlander EE, Gaza EJ, Winget M. Impact of mind-body medicine professional skills training on healthcare professional burnout. Glob Adv Health Med. 2020;9:2164956120906396. 32082951 10.1177/2164956120906396 PMC7005968

[17] Zou L, Sasaki JE, Wei GX, Huang T, Yeung AS, Neto OB, et al. Effects of mind(-)body exercises (Tai Chi/Yoga) on heart rate variability parameters and perceived stress: a systematic review with meta-analysis of randomized controlled trials. J Clin Med. 2018;7(11):404. 30384420 10.3390/jcm7110404 PMC6262541

[18] Chan A-W, Tetzlaff JM, Altman DG, Laupacis A, Gøtzsche PC, Krleža-Jerić K, et al. SPIRIT 2013 statement: defining standard protocol items for clinical trials. Ann Intern Med. 2013;158(3):200–7. 23295957 10.7326/0003-4819-158-3-201302050-00583 PMC5114123

[19] Eldridge SM, Chan CL, Campbell MJ, Bond CM, Hopewell S, Thabane L, Lancaster GA. CONSORT 2010 statement: extension to randomised pilot and feasibility trials. BMJ. 2016;355:i5239. 27777223 10.1136/bmj.i5239 PMC5076380

[20] Levitt HM, Bamberg M, Creswell JW, Frost DM, Josselson R, Suárez-Orozco C. Journal article reporting standards for qualitative primary, qualitative meta-analytic, and mixed methods research in psychology: the APA Publications and Communications Board task force report. Am Psychol. 2018;73(1):26. 29345485 10.1037/amp0000151

[21] Creswell JW, Clark VLP. Designing and conducting mixed methods research. 3rd ed. Los Angeles: Sage publications.; 2017.

[22] Creswell JW, Plano Clark VL, Gutmann ML, Hanson WE. Advanced Mixed Methods Research Designs. In: Tashakkori A, Teddlie C, editors. Handbook of Mixed Methods in Social and Behavioral Research. Thousand Oaks: Sage; 2003. p. 209–240.

[23] Ivankova NV, Creswell JW, Stick SL. Using mixed-methods sequential explanatory design: from theory to practice. Field Methods. 2006;18(1):3–20.

[24] Grohmann A, Kauffeld S. Evaluating training programs: development and correlates of the Questionnaire for Professional Training Evaluation. Int J Train Dev. 2013;17(2):135–55.

[25] Maslach C, Jackson SE. The measurement of experienced burnout. J Organ Behav. 1981;2(2):99–113.

[26] Büssing A, Perrar K. Burnout measurement. Study of a German version of the Maslach Burnout Inventory (MBI-D). Pflege Zeitschrift. 1994;47(3):20–30. 8004334

[27] Neubach B, Schmidt KH. Gütekriterien einer deutschen Fassung des Maslach Burnout Inventory (MBI—D)—Eine Replikationsstudie bei Altenpflegekräften. Zeitschrift für Arbeits-und Organisationspsychologie. 2000;44(3):140–56. 10.1026//0932-4089.44.3.140.

[28] Knispel J, Wittneben L, Slavchova V, Arling V. Skala zur Messung der beruflichen Selbstwirksamkeitserwartung (BSW-5-Rev). Zusammenstellung sozialwissenschaftlicher Items und Skalen (ZIS). 2021. 10.6102/zis303.

[29] Lennartsson AK, Jonsdottir I, Sjors A. Low heart rate variability in patients with clinical burnout. Int J Psychophysiol. 2016;110:171–8. 27535344 10.1016/j.ijpsycho.2016.08.005

[30] Kim HG, Cheon EJ, Bai DS, Lee YH, Koo BH. Stress and heart rate variability: a meta-analysis and review of the literature. Psychiatry Investig. 2018;15(3):235–45. 29486547 10.30773/pi.2017.08.17 PMC5900369

[31] Kirk U, Axelsen JL. Heart rate variability is enhanced during mindfulness practice: a randomized controlled trial involving a 10-day online-based mindfulness intervention. PLoS One. 2020;15(12):e0243488. 33332403 10.1371/journal.pone.0243488 PMC7746169

[32] Kuckartz U, Rädiker S. Qualitative Inhaltsanalyse. Methoden, Praxis, Computerunterstützung: Beltz Verlagsgruppe. Weinheim, Germany; 2018.

[33] Tickle-Degnen L. Nuts and bolts of conducting feasibility studies. Am J Occup Ther. 2013;67(2):171–6. 23433271 10.5014/ajot.2013.006270 PMC3722658

[34] Teresi JA, Yu X, Stewart AL, Hays RD. Guidelines for designing and evaluating feasibility pilot studies. Med Care. 2022;60(1):95–103. 34812790 10.1097/MLR.0000000000001664 PMC8849521

