# Supplementary material for: Feasibility of a custom-tailored, evidence-based, theory-informed, intervention to prevent burnout and reduce stress for healthcare professionals: protocol for a single-arm trial
Source: Pilot Feasibility Stud. 2024 Nov 7;10:134. doi: 10.1186/s40814-024-01553-w (PMC11542239; doi:10.1186/s40814-024-01553-w)
Supplement: Supplementary file 1 — Additional file 1: Table S1. Trial outcomes and modification criteria-basis for the discussion regarding potential modifications for the future pragmatic trial. The table defines success criteria for intervention outcomes which will serve as a basis for the discussion. [file 40814_2024_1553_MOESM1_ESM.docx]

**Additional file 1**

Table 1. Trial outcomes and success criteria-basis for the focus group workshop regarding potential modifications for the future pragmatic trial

| **Objectives** | **Method of assessment** | **Continue without modification** |
| --- | --- | --- |
| **Aim 1: Trial feasibility and acceptability** |  |  |
| Objective 1.1 to measure the recruitment rates of participants | Recording number (*n*) of participants who agreed to participate and number recruited per month | Target of *n* = 30 participants recruited in 2 months (approx. 15/month) |
| Objective 1.2: to measure the attrition of participants | Recording number (n) of participants dropped out over study phase | Drop out rate  ≤ 80% |
| Objective 1.3: to measure the completeness of data collection of participant-reported outcome measures at baseline and post-intervention | Recording number of completed surveys returned to trial team at each time point | ≥ 80% outcome data collected at baseline and post-intervention, respectively |
| Objective 1.4: to determine the feasibility and acceptability of the assessment process | Participants: quantitative survey data | participants rate quantitative survey questions (s. outcomes) with a mean of ≥ 4 |
| Objective 1.5: to determine the protocol adherence | Protocol checklists | ≥ 70% of intervention components carried out according to protocol |
| Objective 1.6: to measure the intervention adherence | Recording number of attended sessions | adherence rate  ≥ 80% (number of sessions attended divided by number of sessions prescribed) |
| Objective 1.7: to determine the feasibility and acceptability of utilising electrophysiological measures | % of electrophysiological measures returned to research staff with valid data  Quantitative survey data | •  ≥ 70% of electrophysiological data collected at pre- and post-intervention  • Participants state that they believe that electrophysiological measures are feasible and acceptable to use as a physiological outcome measure in future definitive trial |
| **Aim 2: Intervention feasibility and acceptability** |  |  |
| Objective 2.1: to determine intervention acceptability, usefulness, participants´ learning, implementation and transfer | Participants: quantitative data (Q4TE)^1^ | Participants´ mean score on Q4TE ≥ 6 |
| Objective 2.2: to determine the safety of the LAGOM program | Recording number of serious adverse events | Participants report no serious intervention-related adverse events |
| Objective 2.3: to determine the perceived fit of the LAGOM program to participants | Participants: quantitative survey data | Participants rate quantitative survey questions (s. Outcomes) with 6 or higher and indicate that they would recommend the program and trainer to other employees |
| Objective 2.4: to determine the perceived benefits, perceived support and barriers to participants | Participants: Semi-structured interviews | Thematic analysis identifies that trial participants perceived the intervention as beneficial and useful, felt supported and did not encounter major barriers in semi-structured interviews |

*Note*. Q4TE=Questionnaire for Professional Training Evaluation
